# Awareness of Thyroid Disorders in Jordan: A National Cross‐Sectional Study

**DOI:** 10.1002/hsr2.71658

**Published:** 2025-12-17

**Authors:** Anas H. A. Abu‐Humaidan, Zain Albdour, Karam Albdour, Diala Al‐Sukhon, Yazan Momani, Nader Alaridah

**Affiliations:** ^1^ Department of Pathology, Microbiology, and Forensic Medicine, School of Medicine The University of Jordan Amman Jordan; ^2^ Faculty of Medicine The University of Jordan Amman Jordan

**Keywords:** education, hypothyroid, Jordan, KAP, Prevention

## Abstract

**Background and Aims:**

Thyroid disorders, despite their prevalence and possible significant complications, remain considerably underdiagnosed. Thus, awareness of thyroid disorders symptoms and risk factors is needed. This study aimed to quantitatively assess the knowledge, attitudes, and practices (KAP) regarding thyroid disorders in Jordan.

**Methodology:**

A cross‐sectional study was conducted to quantitatively assess the KAP of individuals aged 18 and older in Jordan regarding risk factors, symptoms, and treatments of thyroid disorders. A self‐administered electronic questionnaire was developed, validated, and distributed to participants through social media and personal interviews from March to May 2023. The questionnaire was scored to highlight knowledge gaps and analyze demographic factors associated with better KAP scores.

**Results:**

A total of 579 participants completed the survey, with a mean age of 41.4 years, and 62.9% women. The mean knowledge score was 7.2 out of 14.0, indicating moderate awareness. Specific gaps in knowledge relevant to women's health were recognized, where a minority recognized symptoms such as menstrual abnormalities (38.2%), and risk factors such as pregnancy (19.3%). Additionally, a minority believed that thyroid medications should not be stopped during pregnancy (31.8%). Better knowledge correlated with perceiving thyroid disorders as preventable. Poor knowledge was associated with perceiving thyroid disorders as rare and safe. Factors predicting higher knowledge included being female, pursuing medical studies, and having a prior thyroid disorder diagnosis. Most participants preferred visiting an endocrinologist (75.6%) over primary care (19.3%) for thyroid‐related symptoms.

**Discussion:**

This study recommends targeted public health campaigns to enhance awareness of thyroid disorders among women and expectant mothers, and to strengthen the role of primary care centers in disseminating information crucial for creating an informed and proactive population regarding thyroid health.

## 1 Background

Thyroid hormones are crucial for regulating the body's metabolism, growth, and development. Thyroid disorders are among the most common endocrine disorders worldwide, with hypothyroidism being the most common [1]. In iodine‐deficient populations, iodine deficiency is a leading cause of thyroid dysfunction, while in iodine‐replete populations, thyroid autoimmunity is predominant [2]. Despite efforts to increase iodine intake, almost one‐third of the world's population lives in areas of iodine deficiency [3]. In iodine‐replete countries, the prevalence of spontaneous hypothyroidism is between 1% and 2%, while the prevalence of overt hyperthyroidism ranges from 0.2% to 1.3% [4]. Women are particularly more susceptible to developing thyroid disorders, especially with advancing age, during pregnancy, postpartum, and menopause [5].

Thyroid dysfunction encompasses a wide range of manifestations, spanning from subtle, subclinical manifestations to more pronounced and clinically evident symptoms, including fatigue, weight changes, heart rate changes, changes in bowel habits, intolerance to temperature, skin and hair problems, cognitive impairment, tremors, anxiety, depression, and sleep disturbances [4]. Due to their ambiguous nature, these symptoms are commonly overlooked and are attributed to natural aging, so the possibility of thyroid dysfunction is dismissed. Thyroid disorders are associated with various complications if left untreated, including hypertension, hypercholesterolemia, arrhythmias, and vision changes [6, 7].

In Jordan, iodine deficiency has been effectively controlled by the implementation of the Iodine Deficiency Disorders Control Program in 1995 [8]. The overall prevalence of thyroid disorders in Jordan was found to be 11.9%, and the prevalence of undiagnosed thyroid dysfunction was 9% [9]. Similarly, another study in Jordan reported that approximately 10% of participants had a thyroid disorder that went undiagnosed [10]. Both studies had similar findings of undiagnosed thyroid dysfunction, which is higher than estimates of undiagnosed thyroid dysfunction in Europe and the US [11]. This discrepancy highlights the need for awareness campaigns on thyroid disorders in Jordan, which should be guided by an understanding of the current knowledge, attitudes, and practices (KAP) on thyroid diseases.

People who are aware of thyroid disorders and their symptoms are much more inclined to avoid risk factors, to seek medical care when in doubt, and take correct measures related to treatment. Several studies examining the KAP on thyroid disorders have been conducted in India [12], China [13], and Pakistan [14] with a focus on women and university students. Studies conducted in neighboring countries like Saudi Arabia reported varying levels of KAP regarding thyroid disorders [15, 16, 17].

Given the high prevalence of undiagnosed thyroid dysfunction in Jordan and the limited research on the community's KAP regarding thyroid disorders, this study aimed to quantitatively assess the current KAP levels to identify knowledge gaps that may contribute to delayed diagnosis and treatment and to determine factors associated with better knowledge. These findings are essential for informing health policies and designing targeted interventions to improve awareness and early detection.

## 2 Methodology

### 2.1 Study Design and Population

This was a cross‐sectional study to quantitatively assess the KAP of any individual aged 18 and older who resided in Jordan, regarding risk factors, symptoms, and treatments of thyroid disorders, using an online questionnaire.

The minimum representative sample size was determined using an online version of Cochran's formula. A 95% confidence interval and a 5% acceptable margin of error were used in the calculation, along with an estimated proportion of 0.5, resulting in a sample size of 385 participants. The study recorded responses from a total of 622 individuals, of which 579 met the eligibility criteria and were included in the final analysis.

### 2.2 Ethical Approval

The study protocol obtained the IRB (Institutional Review Board) approval at The University of Jordan (Decision Number 16‐2023). Participation in the study was voluntary, and all participants provided electronic consent. Participants were made aware of the anonymous and confidential nature of their involvement before proceeding with the questionnaire.

### 2.3 Questionnaire Development and Pretesting

The questionnaire was constructed via a standardized methodology, which included a literature review, team discussion, evaluation of the questionnaire by experts in the field, and a pilot sample. The questionnaire was initially written in English, then it was translated to Arabic and back‐translated to English, using an adaptation of the back‐translation technique developed by Brislin [18]. *The Checklist for Reporting Results of Internet E‐Surveys (CHERRIES)*, and other relevant EQUATOR guidelines [19] were used in the development of the questionnaire and reporting of the results.

The questionnaire consisted of 34 questions, including true‐false, multiple‐choice checklists, and 5‐point Likert scale statements. The first page of the questionnaire contained informed consent, as well as a description of the questionnaire objectives, and a statement assuring the confidentiality of the collected information. The questionnaire comprised four sections: (1) Sociodemographic details about the participants which included sex, age, occupation, highest level of education, smoking, nationality, household income, social status, and residence; (2) Knowledge about thyroid hormones, as well as risk factors and symptoms of thyroid disorders; (3) Awareness of the treatments of thyroid disorders; (4) Perceptions toward thyroid disorders and sources of information regarding thyroid disorders. The assessment tool can be found in the supporting material (Assessment tool, Supporting Information).

Content validation of the questionnaire was performed by a panel of three medical doctors, one endocrinologist, and two general practitioners, who reviewed the items to ensure comprehensive coverage of key aspects of thyroid disorders relevant to patient awareness. Additionally, a pilot study involving 20 participants, recruited directly by the research team, was conducted to evaluate the questionnaire's face validity and internal consistency. The pilot confirmed the clarity and readability of the questions, supporting face validity. Internal consistency reliability was assessed using Cronbach's alpha, yielding a coefficient of 0.809, indicating good reliability of the questionnaire.

### 2.4 Questionnaire Administration

The questionnaire was uploaded to Google Forms and distributed through multiple channels, including social media platforms such as WhatsApp and Facebook, as well as by directly approaching participants at shopping centers, through a team of medical students. Participation was voluntary and anonymous, with no incentives offered. The first page of the questionnaire clearly explained the voluntary nature of the study and assured participants of their anonymity. To ensure response validity, participants were required to use a verifiable email address, allowing only one response per email; however, email addresses were not recorded. Data collection took place over approximately 1 month, from March 26th to May 1st, 2023.

### 2.5 Questionnaire Scoring

A knowledge scoring system was constructed along with the experts' content validation of the questionnaire, and used consistently for statistical analysis. If a question had only one correct answer, then a correct answer was given a score of 1, and an incorrect answer received a score of 0. For questions with multiple correct answers in a checklist, a correct answer received a score of 0.25, and an incorrect answer received a score of 0. This scoring, instead of a binary one for checklists, ensured that symptoms and risk factors checklists' (24 correct answers in total) would reward partial knowledge without overshadowing the rest of the questionnaire.

### 2.6 Statistical Analysis

Statistical analysis was carried out using IBM SPSS Statistics version 26. Categorical variables were presented as percentages, while continuous variables such as age and score were presented as the mean (± standard deviation). A prespecified analysis to identify demographic variables associated with participants' knowledge of thyroid disorders was conducted using a Pearson Chi‐square (χ^2^) at a 95% confidence interval [20]. Additionally, univariate analysis followed by multivariable logistic regression analysis was conducted to address potential confounding factors and examine the associations between different variables and knowledge scores, variables with a p‐value less than 0.1 in the univariate analysis were included in the multivariate logistic regression model [21]. The results were presented as an adjusted odds ratio (AOR) and a 95% confidence interval. A *p*‐value < 0.05 was considered statistically significant.

## 3 Results

### 3.1 Sociodemographic Characteristics of Participants

Five hundred and seventy‐nine (579) participants met the inclusion criteria and completed the questionnaire. The results revealed a mean knowledge score of 7.22 (±2.86) out of 14 points, indicating overall fair knowledge among the participants. A score lower than 7.25 was assigned as *poor knowledge* while a score of 7.25 or higher was assigned as *good knowledge*.

Most of the participants were female (62.9%) and married (68.7%). The mean age of the participants was 41.4 (±13.2), with the highest percentage being in the age groups of 26–40 (31.1%) and 41–55 (39.7%). As for the participants' educational level, most reported having a bachelor's degree (60.6%), followed by higher education (22.5%) and high school (0.5%). Nearly half of the participants were employed (49.4%), and most reported having health insurance (83.6%) and being non‐smokers (71.7%). About a quarter of the participants (26.4%) had studied or were current students in a medical field like medicine, nursing, pharmacy, dentistry, and physical therapy (Table 1).

**Table 1:** Sociodemographic characteristics of participants.

Variables	Categories	Responses *n* (%)			*p* value*
		Overall	Knowledge level		
			Good	Poor	
			288 (49.7%)	291 (50.3%)	
Gender	Male	215 (37.1)	81 (37.7)	134 (62.3)	<0.001
	Female	364 (62.9)	207 (56.9)	157 (43.1)	
Age	18–25	94 (16.2)	54 (57.4)	40 (42.6)	0.280
	26–40	180 (31.1)	96 (53.3)	84 (46.7)	
	41–55	230 (39.7)	110 (47.8)	120 (52.2)	
	> 55	75 (13.0)	34 (45.3)	41 (54.7)	
Marital Status	Single	157 (27.1)	83 (52.9)	74 (47.1)	0.039
	Married	398 (68.7)	199 (50.0)	199 (50.0)	
	Divorced/Widowed	24 (4.1)	6 (25.0)	18 (75.0)	
Education level	High school or less	98 (16.9)	38 (38.8)	60 (61.2)	0.010
	Bachelor's degree	351 (60.6)	175 (49.9)	176 (50.1)	
	Higher education	130 (22.5)	76 (58.5)	54 (41.5)	
Employment status	Student	77 (13.3)	43 (55.8)	34 (44.2)	0.215
	Employed	286 (49.4)	148 (51.7)	138 (48.3)	
	Unemployed	115 (19.9)	55 (47.8)	60 (52.2)	
	Retired	101 (17.4)	42 (41.6)	59 (58.4)	
Family monthly income in USD**	0–353	122 (21.1)	59 (48.4)	63 (51.6)	0.307
	354–705	138 (23.8)	60 (43.5)	78 (56.5)	
	706–1056	94 (16.2)	54 (57.4)	40 (42.6)	
	1057–1410	85 (14.7)	42 (49.4)	43 (50.6)	
	> 1410	140 (24.2)	73 (52.1)	67 (47.9)	
Health insurance	No	95 (16.4)	35 (36.8)	60 (63.2)	0.006
	Yes	484 (83.6)	253 (52.3)	231 (47.7)	
Smoker	No	415 (71.7)	215 (51.8)	200 (48.2)	0.114
	Yes	164 (28.3)	73 (44.5)	91 (55.5)	
Studied/currently studying a major related to medicine	No	426 (73.6)	164 (38.5)	262 (61.5)	< 0.001
	Yes	153 (26.4)	124 (81.0)	29 (19.0)	
Governorate/place of residence	Central (Amman)	334 (57.7)	163 (48.8)	171 (51.2)	0.785
	Northern	214 (37.0)	108 (50.5)	106 (49.5)	
	Southern	31 (5.3)	17 (54.8)	14 (45.2)	
Diagnosed with a thyroid disorder	No	476 (82.2)	219 (46.0)	257 (54.0)	< 0.001
	Yes	103 (17.8)	69 (67.0)	34 (33.0)	
Family member diagnosed with a thyroid disorder	No	196 (33.9)	77 (39.3)	119 (60.7)	< 0.001
	Yes	383 (66.1)	211 (55.1)	172 (44.9)	
Had any thyroid examination	No	246 (42.5)	96 (39.0)	150 (61.0)	< 0.001
	Yes	333 (57.5)	192 (57.7)	141 (42.3)	

* Pearson's chi‐squared test at a 95% confidence interval assessed differences between participants in the good and poor knowledge groups.

** The income data was collected in Jordanian Dinars and recalculated to United States Dollars (USD) based on the average exchange rate during the study period.

Approximately a fifth of the participants (17.8%) had received a prior diagnosis of a thyroid disorder, and around two‐thirds of the participants (66.1%) had a family member previously diagnosed with a thyroid disorder. More than half of the participants had undergone a physical examination of their thyroid (57.5%).

We observed that females, individuals with higher education, those with health insurance, students pursuing medical‐related majors, and those with a personal or family history of thyroid disorder tended to possess a significantly higher level of knowledge in their respective demographic groups (Table 1).

### 3.2 Knowledge of Thyroid Disorders, Their Risk Factors, and Symptoms

Firstly, participants displayed good knowledge of thyroid function, with most correctly identifying that thyroid hormones affect metabolism (73.6%) and that thyroid hormone plays a role in child development (62.3%). However, less than half of the participants correctly identified that thyroid hormones influence cholesterol levels (40.8%), (Supplementary Table 1).

As for risk factors of thyroid disorders, the most chosen answers were having a family history of thyroid disease (68.7%) and having insufficient or excess iodine in the diet (54.2%). While a minority of participants knew of risk factors such as taking certain medications/drugs (33.9%), radiation exposure (29.4%), and smoking (27.1%). Recent infection was the least reported response (8.1%), and most participants correctly identified that thyroid disorders are not contagious (97.3%) (Supplementary Table 1).

Importantly, most participants identified some of the common symptoms of thyroid disorders such as weight loss/gain (82.0%), fatigue (74.6%), and neck swelling (67.0%), but missed some important ones like heat or cold intolerance (44.7%) menstrual abnormalities (38.2%), palpitations (23.1%) and diarrhea/constipation (26.1%) (Supplementary Table 1).

### 3.3 Knowledge of the Treatment of Thyroid Disorders

In general, participants displayed good knowledge of the available thyroid treatments, and only a minority of participants (15.7%) did not know about, or thought there were no treatments for thyroid disorders, while a smaller percentage (0.5%) held the belief that thyroid disorders should not be treated. Among participants who were aware of the treatability of thyroid disorders (83.8%), medication was the most frequently chosen treatment option (99.7%), followed by surgery (58.7%), and radiation (35.3%). (Supplementary Table 2).

Notably, only 31.8% of participants correctly believed that thyroid medications should not be stopped during pregnancy, and around a third of participants (32.6%) did not know of the side effects of thyroid treatment. When asked whether eating cabbage, cauliflower, and broccoli should be avoided when using thyroid medications, the majority (69.4%) answered in the negative (Supplementary Table 2).

### 3.4 Attitudes and Practices Toward Thyroid Disorders

We assessed how participants perceived the prevalence of thyroid disorders in Jordan. Nearly half of them (48.1%) believed that thyroid disorders were *common* or *very common* in the country. However, only a small percentage considered these disorders to be *very rare* (3.6%) or *rare* (10.2%). (Supplementary Table 3). Then we assessed the participants' beliefs about the danger of thyroid disorders. Interestingly, a significant portion viewed them as either very dangerous (38.7%) or dangerous (23.3%). A considerable number remained neutral (24.9%), while only a small percentage considered having a thyroid disorder to be safe or very safe. Moreover, most participants held the belief that there are preventive measures one can take to avoid developing a thyroid disorder (59.8%) (Supplementary Table 3).

Furthermore, the results indicated that the attitudes of participants were affected by their knowledge, because participants in the *good knowledge* group tended to view thyroid disorders as common, dangerous, and preventable to an extent that is significantly higher than participants in the *poor knowledge* group (Supplementary Table 3).

Assessment of the practices related to thyroid disorders indicated that most of the participants would opt to consult an endocrinologist (75.6%) as their first choice when encountering symptoms of a thyroid disorder, followed by a primary care physician (19.3%). While practices related to information sources on thyroid disorders showed that participants accessed medical information from multiple sources, with friends and family being the most popular (38.5%), followed closely by consulting a physician (35.9%), Google (33.2%), and trusted medical sites (33.3%) (Supplementary Table 4).

### 3.5 Factors Associated With a Better Knowledge Score

A multivariable logistic regression analysis was conducted to address potential confounding factors and examine the associations between different variables and knowledge scores identified in the univariate analysis. The assumptions of multivariate logistic regression, including linearity, absence of multicollinearity, and independence of errors, were met. No significant multicollinearity was detected among predictor variables. The findings of the multivariable logistic regression analysis indicated that being female, possessing a higher level of education, pursuing a field of study related to medicine, and having a previous diagnosis of a thyroid disorder are all significant predictors of possessing good knowledge of thyroid disorders. Conversely, being divorced or widowed, and perceiving thyroid disorders as rare and safe emerged as a significant predictor of having poor knowledge regarding thyroid disorders (Table 2).

**Table 2:** A multivariable logistic regression analysis of factors predicting good knowledge of thyroid disorders.

Variables	Categories	AOR*	95% Confidence Interval	*p* value^ϯ^
Gender	Male	ref		
	Female	1.716	1.089‐2.704	0.020
Marital Status	Single	ref		
	Married	0.978	0.599‐1.596	0.928
	Divorced/Widowed	0.184	0.049–0.694	0.012
Education level	High school	ref		
	Bachelor's degree	1.706	0.958–3.035	0.070
	Higher education	2.392	1.211–4.726	0.012
Health insurance	No	ref		
	Yes	0.623	0.356–1.088	0.096
Study a field related to medicine	No	ref		
	Yes	7.023	4.099–12.034	< 0.001
Personally diagnosed with a thyroid disorder	No	ref		
	Yes	3.243	1.773–5.931	< 0.001
A family member diagnosed with a thyroid disorder	No	Ref		
	Yes	1.143	0.722–1.809	0.568
Been previously physically examined?	No	Ref		
	Yes	1.088	0.684–1.732	0.721
How dangerous do you think thyroid disorders are?	Safe	ref		
	Neutral	2.905	1.509–5.591	< 0.001
	Dangerous	6.361	3.479–11.630	< 0.001
How common do you think thyroid disorders are?	Rare	ref		
	Uncommon	3.175	1.756–5.712	< 0.001
	Common	5.422	3.043–9.66	< 0.001
Do you think steps can be taken to avoid getting thyroid disorders?	No	ref		
	Yes	1.270	0.574–2.808	0.556
	I don't know	0.457	0.200–1.042	0.063

* AOR, adjusted odds ratio.

^ϯ^ Variables with a *p*‐value less than 0.1 in the univariate analysis were included in the multivariate logistic regression model and presented as (AOR) and a 95% confidence interval.

## 4 Discussion

This descriptive cross‐sectional study aimed to assess the KAP on thyroid disorders in Jordan and identify the factors influencing it, addressing a gap in the existing literature on this topic. The findings revealed a moderate level of knowledge, with participants demonstrating good awareness of general thyroid function and its impact on metabolism, but with important gaps in knowledge regarding pregnancy or smoking as risk factors for thyroid disorders, the use of thyroid medication in pregnancy, and the role of primary health care in managing thyroid disorders, highlighting several possible areas for intervention.

Thyroid disorders are globally recognized as a common health concern, yet many go undiagnosed, partially due to the lack of awareness of the risk factors, symptoms, and disease consequences among the general population [22]. Given the high prevalence rates of undiagnosed cases of thyroid disorders in Jordan [9], and the variations in KAP survey results across different communities worldwide [12, 13, 14, 15, 16, 17], it was necessary to assess the knowledge of thyroid disorders among Jordanians. In general, the findings of the study revealed that participants had a fair level of knowledge, as reflected by a mean knowledge score of 7.22 ( ± 2.86) out of 14.00. Similar knowledge scores were observed in the literature [15, 16, 17], however, direct comparisons between studies cannot be made due to disparities in the assessment tools used.

The respondents' place of residence did not significantly impact the KAP score and largely reflected Jordan's population distribution according to the most recent population estimates, with the percentage of this study's participants versus population estimates as the following: Central region: 57.7% versus 63.4%; Northern region: 37.0% versus 28.6%; Southern region: 5.3% versus 8.0%, respectively. Yet the underrepresentation of certain rural areas should be considered when designing targeted public health campaigns. In addition, the low number of participants from the Southern region may have limited the statistical power of the analysis, potentially affecting the generalizability of the findings. The age group did not affect the knowledge score as well, but most of the participants (39.7%) were from the age group (41–55 years of age), probably because of their personal experiences with such disorders making them more motivated to participate, which could have introduced a bias in participation in the study and underrepresentation of older age groups. Nevertheless, this age group was previously found to have a high prevalence of both overt and subclinical thyroid disorders in Jordan [10].

Participants showed good knowledge about the function of the thyroid gland and understood that thyroid dysfunction affects metabolism (73.6%) and that thyroid disorders are not contagious (97.2%), but there was a lack of knowledge regarding the impact of thyroid hormone on cholesterol levels (41.8%). In contrast, previous studies conducted in other countries had higher knowledge of this aspect of thyroid function [23, 24].

In this study, a family history of thyroid disorders was the most recognized risk factor among participants (68.7%), while recent infection was the least recognized (8.1%). A previous study conducted in Saudi Arabia demonstrated a higher level of knowledge of those risk factors compared to our study [15, 24]. Awareness of thyroid disorder risk factors could help in avoiding such risk factors or may result in an earlier diagnosis. Additionally, the study revealed that only 27.1% of participants correctly recognized the association between smoking and thyroid disorders. Given Jordan's high smoking rates [25], and the established link between smoking and hyperthyroidism [26], increasing awareness of this modifiable risk factor could have a direct impact on thyroid health.

Several findings related to women and maternal health stand out from this study, for example, only 19.8% of participants were aware of the association between pregnancy and thyroid disorders, in addition, it was observed that 68.2% of participants either did not know or incorrectly thought women should stop taking thyroid medications during pregnancy, which was similar to a study conducted in India where 63.6% of participants had this understanding [27]. This study also found that only 38.1% of participants were aware that thyroid disorders can lead to menstrual abnormalities, although 62.9% of participants were female, while another study conducted in Saudi Arabia reported better knowledge of this symptom with 48.4% of participants identifying it correctly [24]. It should be noted that menstrual abnormalities were reported by around 30% of hypothyroid patients in a recent study [28], this suggests that this symptom is quite prevalent among hypothyroid patients.

Considering that hypothyroid disorders are estimated to impact around 4%–10% of women, there should be awareness of thyroid disorders symptoms within the realm of women's health [29]. This point was also emphasized in a study among Indian women that concluded that knowledge among women on thyroid disorders was inadequate [12]. Furthermore, untreated thyroid disorders, even subclinical hypothyroidism, during pregnancy can have significant implications for both maternal and fetal health [30, 31]. Raising public knowledge about thyroid disorder risk factors and treatment, especially in pregnant women, is important to improve early detection, ensure proper management, and ultimately enhance maternal and fetal health outcomes.

In this study, the most commonly mentioned symptom for thyroid disorders was weight change (82.0%), closely followed by fatigue (67.0%). On the other hand, diarrhea/constipation was among the less frequently indicated symptoms (26.1%) among participants. These outcomes are consistent with earlier research that reported similar percentages [15, 23, 24]. Awareness of the symptoms of thyroid disorders and adopting appropriate health‐seeking behavior could play a crucial role in reducing the prevalence of undiagnosed cases.

Dietary misconceptions were also prevalent, as 69.4% of participants did not believe that cruciferous vegetables and soy products should be avoided, which exceeded the results of a previous study conducted in India [12], but was comparable to those of a separate study conducted in Saudi Arabia [15]. Cruciferous vegetables and soya products contain naturally occurring goitrogens that have the potential to interfere with thyroid function [32]. Literature on this topic indicates that such foods, consumed at normal levels, do not affect thyroid function in healthy individuals and are unlikely to have an effect in individuals susceptible to thyroid disorders [32].

In our study, a mere 6% of participants believed that thyroid disorders could be treated with herbal medicine, which significantly contrasts with studies conducted in India where a higher percentage perceived herbal medicine as a viable treatment option for thyroid disorders [12, 33]. On the other hand, a separate study conducted in Jordan revealed a high prevalence of herbal product utilization, with chronic disease being the most common reason for their use [34]. While herbal medicine is commonly utilized for general chronic conditions in Jordan, its specific effectiveness in treating thyroid disorders is not widely recognized or supported, probably due to the high success rate and availability of thyroid treatments [35].

It was interesting to note that participants who displayed *good knowledge* were the ones who considered thyroid disorders as common and dangerous, which could be attributed to a previous personal encounter with the disease. Indeed, we found that a staggering 66.1% of participants had a family member who was previously diagnosed with a thyroid disorder. Another reason could be the education received regarding thyroid disorders from physicians, which is supported by the fact that around 35.9% chose to learn about thyroid disorders from a physician, and more than half of the participants (57.5%) reported having undergone a physical examination of their thyroid. The belief that thyroid disorders are preventable was held by most participants (59.8%) and was also more prevalent among participants with *good knowledge*. Preventive measures that could be taken to reduce the risk of thyroid disorders include sufficient iodine intake, exercise, and avoiding smoking [36].

Participants displayed good health‐seeking behavior since they primarily relied on healthcare professionals or their family and friends as their main sources of information about thyroid disorders. This emphasizes the importance of incorporating patient perspectives and social support networks in delivering comprehensive care for thyroid disorders by physicians. Yet a majority indicated a preference for consulting endocrinologists as their first choice, and only 19.3% of participants chose to visit a primary health care center. This could indicate an underutilization of the primary healthcare system in Jordan. Collaborative efforts between primary healthcare providers and specialized practitioners, such as endocrinologists, could lead to more efficient and effective management of thyroid disorders, optimizing resource allocation within the healthcare system [37].

A higher knowledge score was associated with factors such as being female, having higher education, and a personal history of thyroid disease, some of those factors were reported to be associated with better knowledge in several studies in Saudi Arabia [15, 38] and Pakistan [14]. While another study, also in Saudi Arabia, found that those factors did not affect knowledge or perceptions of the disease [16]. In KAP studies among the public in Jordan, being female was often associated with a better knowledge score [39, 40], on the other hand, gender was not a significant factor when KAP studies were performed among university students [41, 42]. Our study identified an additional factor that was significantly associated with knowledge levels, specifically, being divorced or widowed was a significant indicator of having lower knowledge levels regarding thyroid disorders. One possible explanation is that spouses often function as in‐home caretakers, supporting and assisting in managing health‐related matters.

### 4.1 Limitations

The data generated in this study should be interpreted considering some limitations. Firstly, the use of convenience sampling introduced a potential bias regarding participation, which would restrict the generalizability of findings. For example, there was a potential overrepresentation of educated individuals and those with prior experience with thyroid disorders. Conversely, an underrepresentation of rural areas in Jordan ‐especially in the Southern region‐ as well as non‐educated populations, and adults over 55 years of age, could have skewed the results. Secondly, participants' ability to accurately remember and report past experiences, such as previous physical examinations for thyroid disorders or family history of thyroid conditions, may be subject to memory distortions or forgetfulness.

### 4.2 Strengths

This study stands out for its large, diverse sample and comprehensive questionnaire, covering symptoms, risk factors, treatment misconceptions, and healthcare‐seeking behaviors. With participants from multiple regions, the findings closely reflect the broader population in Jordan.

### 4.3 Policy Implication

Integrating thyroid knowledge into maternal care programs and enhancing the training of general practitioners in primary care settings should be central objectives of nationwide thyroid awareness campaigns. These measures are expected to promote early detection and proper management of thyroid disorders, minimize unnecessary referrals to specialists, and improve the accessibility and quality of thyroid care across the population. Further research should assess the effectiveness of such awareness campaigns in promoting early diagnosis and management of thyroid disorders, particularly among women of childbearing age.

Additionally, evaluating thyroid awareness in rural populations, who were possibly underrepresented in this study and are at higher risk of having undiagnosed thyroid dysfunction, is necessary. This could be done by incorporating stratified random sampling or quota sampling to achieve greater representativeness in future studies.

## 5 Conclusion

This study was the first to assess KAP toward thyroid disorders in Jordan, and found clear gaps in recognizing some symptoms and risk factors, especially those related to women's health, like menstrual abnormalities and pregnancy, respectively, and the importance of continuing thyroid treatment during pregnancy. Additionally, many participants preferred seeing an endocrinologist over a primary care doctor for thyroid symptoms. To address those issues, this study recommends that public health campaigns integrate thyroid education into maternal and community healthcare programs to foster better awareness and proactive management of thyroid health, and advises that future research include populations that were underrepresented in this study, like those living in rural areas.

## Author Contributions

**Anas H. A. Abu‐Humaidan:** conceptualization, methodology, data curation, supervision, writing – review and editing, project administration, writing – original draft, validation. **Zain Albdour:** methodology, validation, project administration, data curation, investigation, writing – original draft, writing – review and editing. **Karam Albdour:** visualization, validation, methodology, formal analysis, investigation, writing – original draft. **Diala Al‐Sukhon:** resources, data curation, methodology, validation, investigation, conceptualization. **Yazan Momani:** formal analysis, validation, investigation, writing – original draft, methodology, data curation. **Nader Alaridah:** writing – review and editing, writing – original draft, project administration, supervision, methodology.

## Funding

The authors received no specific funding for this work.

## Conflicts of Interest

The authors declare no conflicts of interest.

## Transparency Statement

The lead author Anas H. A. Abu‐Humaidan affirms that this manuscript is an honest, accurate, and transparent account of the study being reported; that no important aspects of the study have been omitted; and that any discrepancies from the study as planned (and, if relevant, registered) have been explained.

## Supporting information

Assessment tool, Supplementary Material.

**Supplementary Table 1:** Knowledge of thyroid function and thyroid disorders. **Supplementary Table 2:** Knowledge of the treatment of thyroid disorders. **Supplementary Table 3:** Participants' attitudes toward thyroid disorders. **Supplementary Table 4:** Practices toward thyroid disorders.

## Data Availability

The data that support the findings of this study are available from the corresponding author upon reasonable request. The authors confirm that the data supporting the findings of this study are available within the article. The raw data used in generating these results will be made available from the authors upon request.
