# Supplementary material for: Awareness of Thyroid Disorders in Jordan: A National Cross‐Sectional Study
Source: Health Sci Rep. 2025 Dec 17;8(12):e71658. doi: 10.1002/hsr2.71658 (PMC12710433; doi:10.1002/hsr2.71658)
Supplement: Supplementary file 1 — Assessment tool, Supplementary Material. [file HSR2-8-e71658-s002.pdf]

Knowledge, Awareness, and Perceptions of the Jordanian Population Towards Thyroid Disorders

Dear participant,

We are a research group from the School of Medicine at The University of Jordan investigating the knowledge, awareness, and perceptions of thyroid disorders among Jordanian adults (above 18 years of age).

We invite you to participate in this survey which will only take a few minutes (around 4-6 minutes). Please note the following:

*Your participation is voluntary, and you have the right to opt out of this questionnaire at any given moment.

*This survey was approved by the Institutional Review Board which is the reference body for scientific research ethics at The University of Jordan.

*Your participation is valuable and will help guide public health policies toward thyroid diseases and eventually help thyroid patients.

*Email verification is required for the authentication of unique participants and will not be shared with any third party.

*Your personal information will be anonymized and will not be shared with any people other than the researchers working on data analysis.

*If you have any questions, do not hesitate to contact us at thyroidju2022@gmail.com

* Indicates required question

1. I agree to participate in this survey *

Mark only one oval.

- ◯ Yes
- ◯ No

Demographics

2. Age *

3. Gender *

Mark only one oval.

- Male
- Female

4. Social status *

Mark only one oval.

- Single
- Married
- Divorced

5. Nationality *

Mark only one oval.

- Jordanian
- Palestenian
- Syrian
- Other
- I'd rather not say

6. Do you currently live in Jordan *

Mark only one oval.

- Yes
- No

7. Governorate *

Mark only one oval.

- Irbid
- Albalqa
- Jarash
- Alzarqa
- Altalifeh
- Ajloun
- Alaqaba
- Amman
- Karak
- Madaba
- Maan
- Mafraq

8. Level of education *
The most recent degree you have completed (not your current one)

Mark only one oval.

- High school or less
- Bachelor's degree
- Higher education

9. Employment status *

Mark only one oval.

- Employed
- Unemployed
- Student

10. Monthly household income *

Mark only one oval.

- 0-250 JDs
- 251-500 JDs
- 501-750 JDs
- 751-1000 JDs
- 1001+ JDs

11. Do you have health insurance? *

Mark only one oval.

- Yes
- No

12. Do you have/are you currently studying for a medical related degree? *
(Medicine, dentistry, rehabilitation, nursing, Doctor of Pharmacy)

Mark only one oval.

- Yes
- No

13. Are you a smoker? *

Mark only one oval.

- Yes
- No

14. Have you ever been diagnosed with a thyroid disorder? *

Mark only one oval.

- Yes
- No

15. Do you know any relatives/ friends who suffer from a thyroid disorder? *

Mark only one oval.

- Yes
- No

16. Have you undergone any testing for your thyroid gland? *

Mark only one oval.

- Yes
- No

Knowledge of thyroid function and thyroid disorders

17. Does thyroid hormone affect metabolism? *

Mark only one oval.

- Yes
- No
- I don't know

18. Does thyroid hormone affect cholesterol levels? *

Mark only one oval.

- Yes
- No
- I don't know

19. Does thyroid hormone play a role in child development? *

Mark only one oval.

- Yes
- No
- I don't know

20. Thyroid disorders are contagious *

Mark only one oval.

- True
- False

21. Which of the following increase the risk of thyroid disorders? (Check all that apply) *

Check all that apply.

- [ ] Smoking
- [ ] Radiation exposure
- [ ] Insufficient/overexposure to iodine
- [ ] Gender
- [ ] Pregnancy
- [ ] Certain medications/drugs
- [ ] Increased age
- [ ] Family history of thyroid disease
- [ ] Recent infection
- [ ] Chronic sunlight exposure
- [ ] Autoimmune disease

22. What are the signs and symptoms of thyroid disease (Check all that apply) *

Check all that apply.

- [ ] Unintentional weight loss/gain
- [ ] Hair loss
- [ ] Heart problems
- [ ] Excessive feeling of hotness
- [ ] Fatigue and sleepiness
- [ ] Memory problems
- [ ] Constipation/Diaarhea
- [ ] Voice changes
- [ ] Irregular menstrual cycle
- [ ] Neck swelling
- [ ] Depression/anxiety
- [ ] Change in appetite
- [ ] Bulging eyes

Treatment of thyroid disorders

23. Can thyroid disorders be treated? *

Mark only one oval.

- ⬭ Yes
- ⬭ No
- ⬭ Not always
- ⬭ I don't know

24. How can someone with thyroid disease be treated? (Check all that apply) *

Check all that apply.

- [ ] Medications
- [ ] Surgery
- [ ] Radiotherapy
- [ ] Herbal medicine

25. Do thyroid disorder treatments have side effects? *

Mark only one oval.

- ⬭ No
- ⬭ It may have minor side effects (palpitations, nausea, vomiting, loss of taste)
- ⬭ It may have major side effects (severe infections, liver damage, permanent voice change, lung problems)
- ⬭ I don't know

26. If you have a thyroid disorder, you should avoid eating cabbages, cauliflowers, and broccoli *

Mark only one oval.

- ⬭ True
- ⬭ False

27. Thyroid medications should be stopped during pregnancy *

Mark only one oval.

- True
- False
- I don't know

Attitudes and practices towards thyroid disease

28. How common do you think thyroid disorders are in Jordan? *

Mark only one oval.

	1	2	3	4	5	
Very	○	○	○	○	○	Very common

29. How serious do you think thyroid disorders are? *

Mark only one oval.

	1	2	3	4	5	
Not	○	○	○	○	○	Very serious

30. Can people take effective actions to prevent thyroid disease? *

Mark only one oval.

- Yes
- No
- I don't know

31. Who would you first visit if you experience symptoms of a thyroid disorder? *

Mark only one oval.

- Primary care center
- Endocrinologist
- Pharmacist
- Family/friends
- Others
- No one

32. *

What sources of information do you use to obtain information about thyroid disorders?

Mark only one oval.

- Social media
- Official medical websites (WHO, CDC, Ministry of health)
- Consulting a doctor
- Newspaper/magazine
- Friends and Family
- Google

This content is neither created nor endorsed by Google.

Google Forms
