# Supplementary material for: Awareness of Thyroid Disorders in Jordan: A National Cross‐Sectional Study
Source: Health Sci Rep. 2025 Dec 17;8(12):e71658. doi: 10.1002/hsr2.71658 (PMC12710433; doi:10.1002/hsr2.71658)
Supplement: Supplementary file 2 — Supplementary Table 1: Knowledge of thyroid function and thyroid disorders. Supplementary Table 2: Knowledge of the treatment of thyroid disorders. Supplementary Table 3: Participants' attitudes toward thyroid disorders. Supplementary Table 4: Practices toward thyroid disorders. [file HSR2-8-e71658-s001.pdf]

Awareness of Thyroid Disorders in Jordan: A National Cross-Sectional Study

Anas H. A. Abu-Humaidan[1*], Zain Albdour[2], Karam Albdour[2], Diala Al-Sukhon[2], Yazan Momani[2], Nader Alaridah[1]

[1] Department of Pathology, Microbiology, and Forensic Medicine, School of Medicine, The University of Jordan, Amman 11942, Jordan.

[2] Faculty of Medicine, The University of Jordan, Amman 11942, Jordan

*Address correspondence to:

Anas Abu-Humaidan M.D. Ph.D.

E-mail: A.abuhumaidan@ju.edu.jo

Tel. number: +962779227922

Supplementary Tables

Supplementary Table 1. Knowledge of thyroid function and thyroid disorders

**Questions and choices**	**Responses n (%)**
Does thyroid hormone affect metabolism?	
No	11 (1.9)
Yes	426 (73.6)
I don't know	142 (24.5)
Does thyroid hormone affect cholesterol levels?	
No	47 (8.1)
Yes	236 (40.8)
I don't know	296 (51.1)
Does thyroid hormone play a role in child development?	
No	26 (4.5)
Yes	361 (62.3)
I don't know	192 (33.2)
Thyroid disorders are contagious?	
True	16 (2.8)
False	563 (97.2)
Which of the following increases the risk of having a thyroid disorder?	
Smoking	157 (27.1)
Radiation exposure	170 (29.4)
Insufficient/Excess Iodine	314 (54.2)
Gender	136 (23.5)

Pregnancy	112 (19.3)
Certain medications/drugs	196 (33.9)
Increased age	155 (26.8)
Family history of thyroid disease	398 (68.7)
Recent infection	47 (8.1)
Chronic sunlight exposure *	44 (7.6)
Other autoimmune diseases	171 (29.5)
Obesity	269 (46.5)
Which of the following are symptoms of having a thyroid disorder?	
Weight changes	475 (82.0)
Hair loss	274 (47.3)
Palpitations	134 (23.1)
Heat/cold intolerance	259 (44.7)
Fatigue	432 (74.6)
Memory disturbances	197 (34.0)
Diarrhea/Constipation	151 (26.1)
Hoarseness	204 (35.2)
Menstrual abnormalities	221 (38.2)
Neck swelling	388 (67.0)
Anxiety/Depression	286 (49.4)
Appetite changes	331 (57.2)
Proptosis	258 (44.6)

* Not considered a risk factor

Supplementary Table 2. Knowledge of the treatment of thyroid disorders

**Questions**	**Responses n (%)**
Can thyroid disorders be treated	
Yes	485 (83.8)
No treatment is available	14 (2.4)
No treatment is required	3 (0.5)
I don't know	77 (13.3)
What are the treatment methods for thyroid disorders? *	
Medications	484 (99.7)
Surgery	285 (58.7)
Radiation therapy	171 (35.3)
Herbal therapy	35 (7.2)
Do thyroid disorder treatments have side effects? *	
It may have minor side effects (palpitations, nausea, vomiting, loss of taste)	249 (51.3)
It may have major side effects (severe infections, liver damage, permanent voice change, lung problems)	46 (9.5)
No	32 (6.6)
I don't know	158 (32.6)
If you have a thyroid disorder, you should avoid eating cabbages, cauliflowers, and broccoli	
True	177 (30.6)
False	402 (69.4)
All thyroid medications should be stopped during pregnancy	
True	109 (18.8)

False	184 (31.8)
I don't know	286 (49.4)

[*] Participants who responded "Yes" to "Can thyroid disorders be treated?" were able to answer these questions.

Supplementary Table 3. Participants' attitudes toward thyroid disorders

Questions	Choices	Responses n (%)			P value *
		Overall	By knowledge level		
			Good (n=288, 49.7%)	Poor (n=291, 50.3%)	
How common do you think thyroid disorders are in Jordan?	Rare	80 (13.8)	18 (22.5)	62 (77.5)	<0.001
	Uncommon	221 (38.2)	106 (48.0)	115 (52.0)	
	Common	278 (48.0)	170 (61.2)	108 (38.8)	
How dangerous do you think thyroid disorders are?	Safe	76 (13.1)	15 (9.7)	61 (80.3)	<0.001
	Neutral	144 (24.9)	57 (39.6)	87 (60.4)	
	Dangerous	359 (62.0)	219 (61.0)	140 (39.0)	
Do you think steps can be taken to avoid	No	44 (7.6)	29 (65.9)	15 (34.1)	<0.001

getting thyroid disorders?	Yes	346 (59.8)	207 (59.8)	139 (40.2)	
	I don't know	189 (32.6)	58 (30.7)	131 (69.3)	

* Pearson's chi-squared test at a 95% confidence interval assessed differences between participants in the good and poor knowledge groups.

Supplementary Table 4. Practices toward thyroid disorders

**Questions and choices**	**Responses n (%)**
Where would you go if you were suffering from symptoms of thyroid disease?	
Primary care center	112 (19.3)
Endocrinologist	438 (75.6)
Pharmacist	5 (0.9)
Friends/Family	20 (3.5)
Others	4 (0.7)
No one	29 (5)
What sources of information do you use to obtain information about thyroid disorders?	
Consulting a physician	208 (35.9)
Newspapers/magazines	78 (13.5)
Family/friends	223 (38.5)
Social Media (Facebook, WhatsApp)	152 (26.3)
Google	192 (33.2)
Official medical sites	193 (33.3)
